# Lifetime Reduction and Enhanced Emission of Single Photon Color Centers in Nanodiamond via Surrounding Refractive Index Modification

**DOI:** 10.1038/srep11179

**Published:** 2015-06-25

**Authors:** Asma Khalid, Kelvin Chung, Ranjith Rajasekharan, Desmond W.M. Lau, Timothy J. Karle, Brant C. Gibson, Snjezana Tomljenovic-Hanic

**Affiliations:** 1 School of Physics, University of Melbourne, Parkville 3010, VIC, Australia; 2 Department of Electrical and Electronic Engineering, University of Melbourne, Parkville 3010, VIC, Australia

## Abstract

The negatively-charged nitrogen vacancy (NV^−^) center in diamond is of great interest for quantum information processing and quantum key distribution applications due to its highly desirable long coherence times at room temperature. One of the challenges for their use in these applications involves the requirement to further optimize the lifetime and emission properties of the centers. Our results demonstrate the reduction of the lifetime of NV^−^ centers, and hence an increase in the emission rate, achieved by modifying the refractive index of the environment surrounding the nanodiamond (ND). By coating the NDs in a polymer film, experimental results and numerical calculations show an average of 63% reduction in the lifetime and an average enhancement in the emission rate by a factor of 1.6. This strategy is also applicable for emitters other than diamond color centers where the particle refractive index is greater than the refractive index of the surrounding media.

The rapid growth of quantum-information science has driven the research and development of single photon sources (SPSs) in recent years^1^,^2^,^3^,^4^. Robust SPSs have enabled progress in the optical detection, characterization and manipulation of single quantum objects^2^,^3^. SPSs have also found application in high resolution nanoscale imaging^5^,^6^. An ideal SPS should emit on demand single photons with sufficient brightness at an arbitrarily fast rate^1^,^7^. In order to meet these demands, novel nanophotonic designs around emitters are required to engineer their lifetime and emission properties.

Color centers in nanodiamonds (NDs) have been explored extensively due to their photostable single photon emission at room temperature^3^,^8^. The negatively charged nitrogen vacancy (NV^−^) is one of several color centers (or optically active defects) in diamond, which is known for its unique optical properties including room-temperature photostable emission^8^ and high quantum efficiency^9^. The NV^−^ center consists of a substitutional nitrogen atom adjacent to a vacancy in the diamond lattice as pictured in Fig. 1(a)^8^. The NV^−^ center is treated as a three level electronic system having a ground triplet state ^3^A, a triplet excited state ^3^E and an intermediate singlet state ^1^A^8^, as shown in Fig. 1(b). When optically excited with a 532 nm laser, the system decays either radiatively from ^3^E to ^3^A or non-radiatively through the singlet intermediate spin level ^1^A. This optical transition exhibits a zero phonon line at 637 nm accompanied with a vibronic side band in the range of 650–750 nm, as shown in Fig. 1(c). The transition has a high quantum efficiency as compared to other defects in diamond^10^ and quantum dots^11^.

The NV^−^ center, decaying by spontaneous emission is treated as a radiating dipole (inside the high refractive index ND)^12^,^13^,^14^, whose emission is mainly affected by (i) the proximity of the dipole to the dielectric substrate and its refractive index^15^,^16^,^17^, (ii) the encapsulation of the dipole in the (sub-wavelength scaled) ND particle^13^, (iii) the orientation of the NV^−^ dipole with respect to the substrate^16^, and (iv) the refractive index of the cover/top cladding material into which dipole is radiating its energy.

Studies show that for the case of a highly reflecting substrate^16^,^17^, the radiation field is the result of interference of the original dipole field superimposed on the reflected field^18^. The use of a partially reflecting substrate enhances the intensity of dipole radiation above the substrate. Moreover, for a ND encapsulating NV^−^, the dipole is located at a distance, *z*, close to the substrate such that *z* ≪ *λ*, where *λ* is the dipole emission wavelength. This enables reflected evanescent waves to transport their energy and contribute to the radiated power^16^,^19^. The contribution of this effect is greater closer to the surface, i.e., at small *z*. The experimental studies referenced^18^,^19^,^20^,^21^,^22^ and theoretical models^16^,^17^ provide evidence that the spontaneous emission properties can be enhanced by modifying the local environment around the NDs. Engineering the structural properties would also address the important challenge of the optimisation of broad-band fluorescence emission rates^22^,^23^ and longer lifetimes of ND encapsulated NV^−^ centers at room temperature as compared to the bulk diamond^9^.

Generally^24^, the radiation from emitters within NDs are collected after transmission through a transparent glass substrate. This emission is found to be strongly dependent on the ND-glass interface and the optical properties are influenced by effects arising from the refractive index contrast of the ND-glass interface^16^,^17^. A recent study to determine the fluorescence lifetimes of individual NDs, has reported longer lifetimes as compared to NDs on glass substrates^9^. The study was carried out in the presence of a near unity refractive index substrate, which eliminated the influence of the substrate. A longer lifetime implies that the NDs, on their own, radiating in air (*n *= 1) have a correspondingly lower emission rate. Other studies^25^,^26^,^27^,^28^ have explored the angular dependence of collection efficiency, both on the dipole radiation pattern and the lens collection angle. All such experiments to engineer or understand the emission properties of NDs discuss the effects of dielectric interface, encapsulation and collection angle on the emission of NV^−^ dipole as a function of the dipole distance *z* from the substrate^26^. However, the influence of a high refractive index cover material coating the NDs and detection of emitted radiation through the top interface has not been explored so far.

In this manuscript, we present the emission characteristics of NDs on a reflecting silicon (Si) substrate, radiating in air and thin film cover. We study a structure where the NV^−^ dipole is radiating in the presence of finite and planar interfaces and nano-sized diamond encapsulation. We have used a higher refractive index substrate which is crucially also absorbing within the absorption and emission bands of the emitter and the system is probed from above. The ability to efficiently collect the emission is dependent on the dipole orientation. If modification of the dipole’s environment transforms its radiation pattern, this changes the fraction of dipole emission radiated to far field collection optics.

We show here that the lifetime of the dipole and its emission properties are improved by coating the NDs with a polymethyl methacrylate (PMMA) film having a refractive index higher than air. Our work not only provides an insight to the basic physics of the radiating dipole but is also important for several applications requiring brighter SPSs with shorter lifetimes for the realization of practical applications of these SPS in emerging quantum technologies^29^,^30^ and high resolution medical imaging applications^5^,^6^,^30^.

## Methods and Results

**Figure 2:**
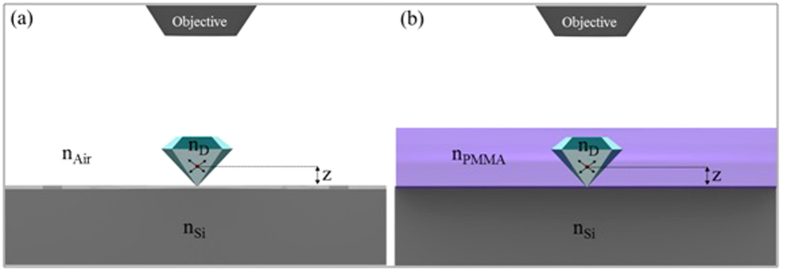
A simplified picture of the sample geometry, showing a single NV^−^ dipole enclosed in the ND, at a distance *z* from the lower ND-Si interface. The dipole’s radiation was detected in the upper (**a**) ND/Air or (**b**) ND/PMMA interface with a 0.95 NA objective lens.

The geometry of the structure under investigation is illustrated in Fig. 2. We experimentally detected the emitted power from NDs seeded on a partially reflecting Si substrate, before and after coating with a thin polymer film of refractive index, higher than air.

The geometry is simplified by considering the fact that the NV^−^ center is radiating into an upper and lower interface, where the upper space constitutes either air, *n*_*Air*_ = 1.0, or PMMA, *n*_*PMMA*_ = 1.48 as shown in Fig. 2(a,b) respectively. The photons radiated from the NV^−^ center were collected from the upper interface with an air objective. For a structured environment like this, the emission of NV^−^ is known to be strongly dependent on the refractive index contrast at the interfaces^18^,^26^,^31^. The spontaneous emission lifetimes and emission count rates for the PMMA coated, ND encapsulated NV^−^ centers were measured. The reduction in lifetime was accompanied by a corresponding increase in the emission rate after polymer coating. The experimentally observed enhancement in emission was verified using numerical modeling with RSoft (Finite Difference Time Domain) and COMSOL (Finite Element Method).

## Experimental Results

**Figure 3:**
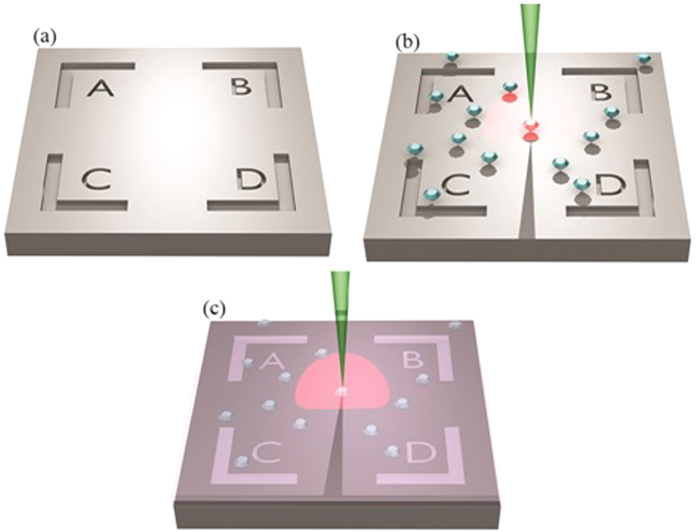
A schematic image depicting the method of sample fabrication and its optical characterization. (**a**) Central region of the FIB marker milled on the Si substrate to identify NDs. NDs seeded in this area were examined for optical properties before and after coating. NDs on Si substrate illuminated with a green laser, radiate a characteristic red fluorescence (**b**) before and (**c**) after PMMA coating.

A Si substrate was marked to address the same centers before and after polymer coating. A marked pattern shown in the schematic of Fig. 3(a) was milled^32^ on the substrate using a focused ion beam (FIB) and had a role to determine the positions of single photon emitters. NDs in a 2 mg/mL aqueous solution were drop cast on the marked Si substrate, as illustrated in Fig. 3(b).

**Figure 4:**
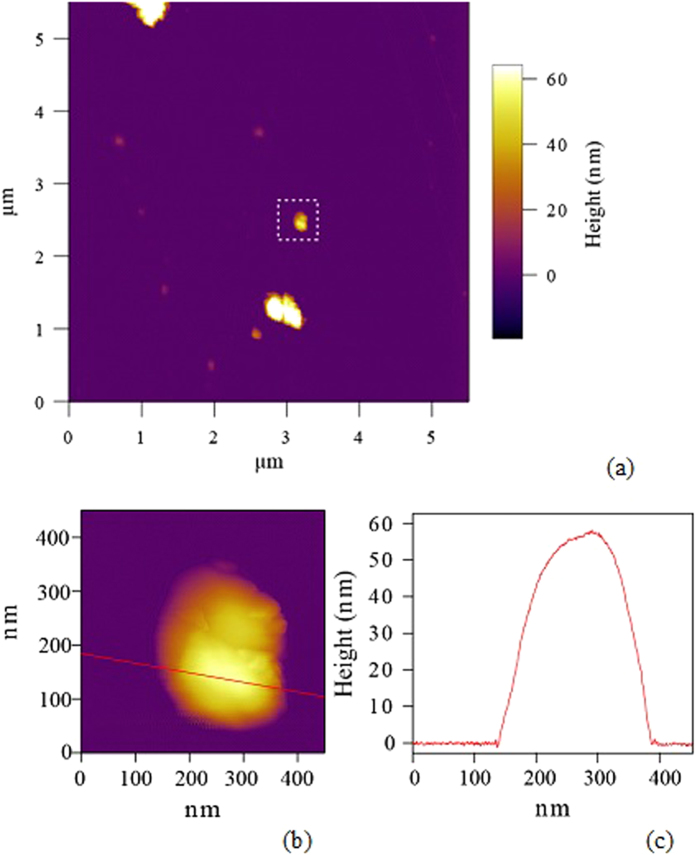
(**a**) AFM image of NDs seeded on Si substrate to measure the average height of NDs before PMMA coating. The ND inside the dotted box contains single NV^−^ center and is representative of the ten NDs characterized in this work. (**b**) A zoomed 500 × 500 nm^2^ image of the selected ND and (**c**) its height from the surface of the substrate are shown.

Before coating the sample with polymer, Atomic Force Microscopy (AFM) measurements were made to obtain the average ND height. Eleven NDs on the marked Si substrate, containing single NV^−^ centers, showed heights in the range of 45–88 nm, averaging to 65 nm.The AFM images for one of the eleven NDs encapsulating the single NV^−^ center are shown in Fig. 4.

**Figure 5:**
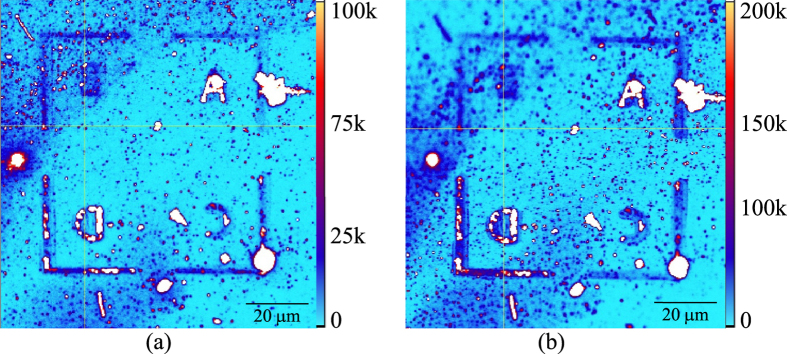
A 100 × 100 μm^2^ confocal scan of the NDs on the Si substrate (**a**) before and (**b**) after PMMA coating. The count rate (counts/s of detector) scale is shown to the right of each figure.

The marked region after ND deposition was optically characterized with an in-house built confocal fluorescence microscope described in Experimental Section. A 100 × 100 μm^2^ confocal fluorescence map of the NDs deposited marked Si substrate before coating is shown in Fig. 5(a).

**Figure 6:**
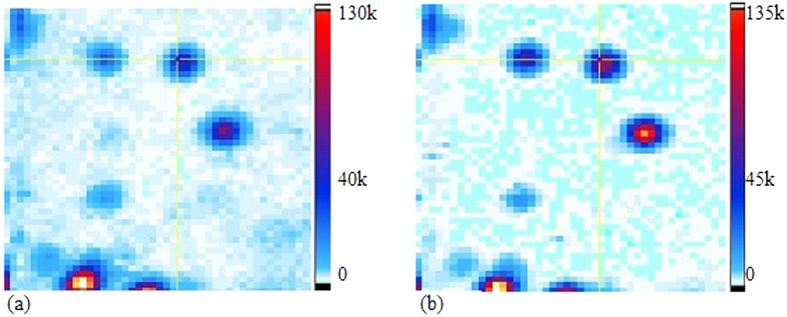
A 5 × 5 μm^2^ confocal scan of the NDs on Si substrate before (**a**) and after (**b**) PMMA coating. The fluorescing ND labeled with the cross hair was checked for the presence of single NV^−^ center.

**Figure 7:**
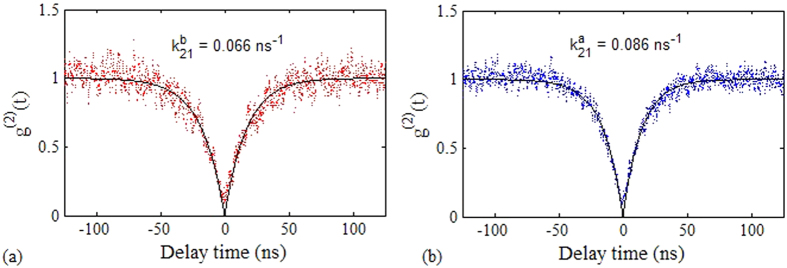
Intensity autocorrelation data as a function of delay time (**a**) before and (**b**) after PMMA coating with a time bin of 128 ps. The data was recorded at 50 μW pump power and fitted with a single exponential function (black curve), indicative of a two-level emitter. The calculated decay rates before and after coating are also provided.

A total of ten emitters on the scan of Fig. 5 were found to exhibit a characteristic minimum in the intensity autocorrelation function at zero time delay *g*^*(2)*^ *(∆t = 0)* → 0, indicative of single photon emission^33^. Emitters that showed a dip in the autocorrelation function *g*^*(2)*^ *(∆t = 0) *≤ 0.2 were selected and studied for the emission properties before and after polymer coating. The emitter labeled with a crosshair in Fig. 6(a) exhibited a characteristic antibunching dip in its intensity autocorrelation function which is shown in Fig. 7(a), at an input power of 50 μW.

Once the ND was identified as containing a single NV^−^ center, we proceeded to measure its fluorescence lifetime *τ*. To do this, the intensity autocorrelation data was recorded at a range of low powers. At each value of power, the data was curve fitted with a single exponential function,


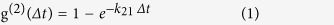


where the fitting parameter *k*_*21*_ is the spontaneous emission decay rate from excited state to ground state^25^,^34^. The lifetime is given by the inverse of the decay rate of the *g*^*(2)*^ *(∆t)* function in the weak excitation limit (or low power limit). The decay factor before coating *k*^*b*^_*21*_ was calculated and plotted as a function of the pump power for each of the selected emitters. The linearly varying decay rate *k*^*b*^_*21*_ versus the incidence power data was fitted using the least squares method and the line of best fit was extrapolated to get the decay rate at zero power, *k*^*b*^_*21*_ (*P *= 0). The intrinsic spontaneous emission lifetime was calculated for the emitters using *τ*^*b*^_*21*_ *= 1 / k*^*b*^_*21*_*(0).*

**Figure 8:**
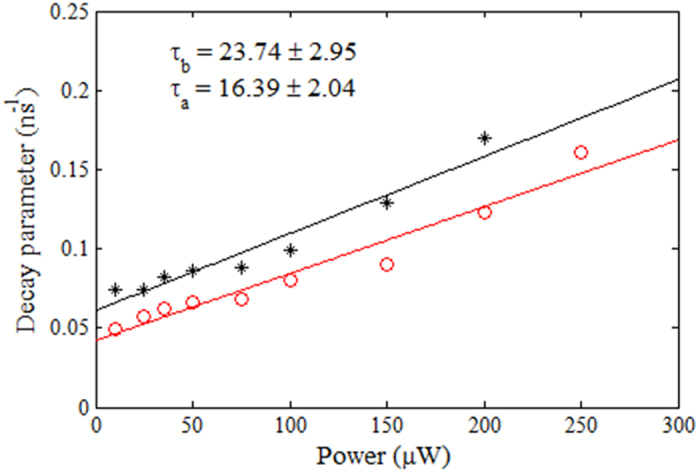
Lifetime calculation of the NV^−^ center encapsulated in ND, labeled with crosshair in Fig. 6(a) before (red) and after (black) PMMA coating.

The lifetime measurement for the ND shown in Fig. 6(a) is presented by the red points in Fig. 8(a). A lifetime of *τ*^*b*^_*21*_ = 23.74 ± 2.95 ns was observed for the emitter under investigation. The procedure was repeated for the ten NV^−^ centers and the spontaneous emission lifetime values, before coating for these single photon emitters, are shown in Table 1.

The emitters were found to have lifetimes in a broad range from ∼ 13 ns up to 29 ns before PMMA coating.

**Figure 9:**
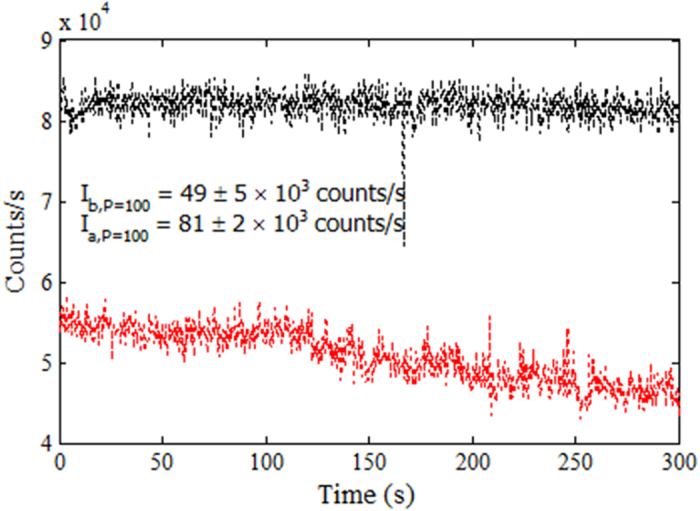
Emission rate for the ND labeled with a crosshair in Fig. 6, before (red points) and after (black points) PMMA coating at 100 μW.

The emission rate (counts/s) of each center before coating *I*_*b*_ was also recorded at a range of different low and high powers (10 μW ≤ P ≤ 1200 μW). The red data in Fig. 9 shows the emission rate of the above mentioned ND recorded for 300 s at a pump power of 100 μW. The average count rate was found to be 49 kcounts/s before PMMA coating.

**Figure 10:**
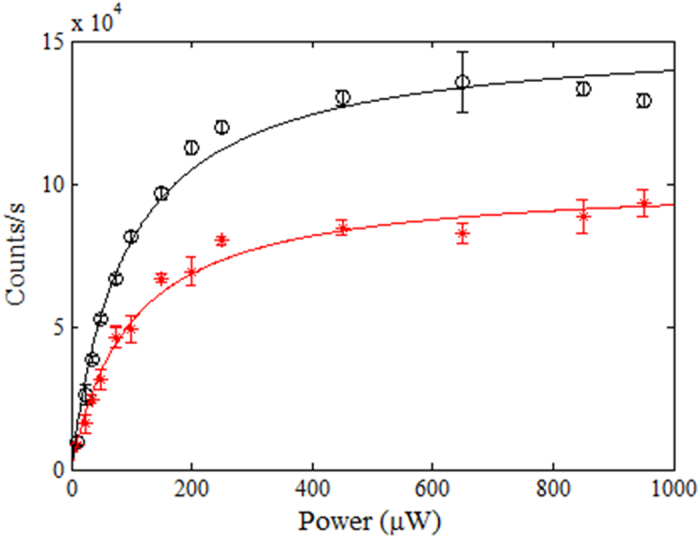
Background subtracted average single photon count rate as a function of excitation power for the ND labeled with cross hair in Fig. 6, before (red) and after (black) PMMA coating. The emission from the NV^−^ center first increases linearly at lower powers and then saturates at higher excitation powers.

The emission saturation curves were recorded to determine the maximum emission rates for the SPSs before coating. Initially, the average emission rate linearly increased with power in the low laser power regime. For higher pump powers, the counts started to saturate^32^,^35^. The saturation curve for the NV^−^ center under discussion (before coating) is shown as red points in Fig. 10.

These count rates, *I*_*b*_ *(P)*, recorded for each emitter at a range of excitation powers *P* before coating was curve fitted with the function^36^,


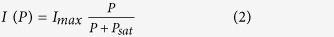


where *I*_*max*_ is the maximum obtainable count rate and *P*_*sat*_ is the power where counts saturate.

After measuring the count rates, lifetimes and saturation curves for the selected NDs, the ND-Si sample was spin coated with PMMA. The 950 PMMA A Resist (MicroChem) was used with 2% of the polymer in anisole solution. The solution was spin coated on ND-Si sample at 2000 rotations per minute (RPM) speed. The refractive index of the PMMA, *n*_*PMMA*_ and thickness, *t* of the resulting spin coated film was measured experimentally with a spectroscopic ellipsometer (SE). The refractive index and thickness were found to be *n*_*PMMA*_ = 1.479 ± 0.001 (at *λ *= 637 nm) and *t *= 111.05 ± 0.03 nm respectively for the PMMA film on ND-Si sample. The schematic of the sample after coating is illustrated in Fig. 3(c).

The optical emission properties for the ten NDs, shown in Fig. 5(b) and tabulated in Table 1, were analyzed again after PMMA coating. Confocal microscopy was used to scan the same area of the marked substrate after coating and FIB markers located the exact positions of the single NV^−^ centers. Figure 6(b) shows a zoomed 5 × 5 μm^2^ window, image of the ND after coating. The surroundings of the ND were unchanged after spin coating as compared to those before coating (Fig. 6(a)). However, the maximum counts of the confocal fine scan increased after coating, as evident from the brightness of the scan and the intensity scale bar to the right of each image.

The antibunching data for each ND was recorded again at a range of input power values after PMMA coating. All NDs were found to exhibit single photon emission after PMMA coating, which means that the PMMA coating did not add significant background fluorescence to the emission from the NV^−^ center. For every ND, the autocorrelation data at each power was compared before and after coating. Figure 7(b) shows the *g*^*(2)*^ *(∆t)* fit for the coincident counts of the ND recorded at *P *= 50 μW after coating. The dip around *∆t *= 0 ns was steeper and narrower after coating which corresponds to a faster decay rate *k*^*a*^_*21*_ to the ground state at 50 μW. A similar trend was observed for the *g*^*(2)*^ *(∆t)* function at each value of power. The decay rates were plotted as a function of power as shown in Fig. 8 (black data points).

A linear fit and extrapolation of the data provided the value of intrinsic spontaneous emission lifetime for the emitter after PMMA coating as *τ*^*a*^_*21*_ *= 1 / k*^*a*^_*21*_*(0) *= 16.39 ± 2.04 ns. This means that the lifetime of the emitter was reduced by a factor 0.69 ± 0.12 after coating with PMMA. For all other emitters the reduction in the lifetime after coating was in a range of a factor 0.48–0.89.

The emission rates for each ND were recorded again at a range of different powers after coating. Figure 9 shows an effective increase in the emission rate at P = 100 μW for the ND after coating (black data points) as compared to the uncoated data (red points). The saturation counts curve is also higher for the ND as shown with black data points in Fig. 10. The background subtracted average emission rate was measured for the center at each power and the increase in counts after coating was observed for each value of power. The enhancement in counts was plotted against the input laser power as shown in Fig. 10 (black data points). The graph shows an enhancement of around 1.5 times for this particular ND. The increase in counts corresponds to an approximately equal decrease in lifetime which is consistent with the literature^24^.

The lifetime data and emission rate measurements were carried out for all the selected single photon emitters after coating. For other emitters the increase in the emission rate after coating ranged between 1.1−2.1. The data shown in Table 1 statistically demonstrates the lifetime reduction and emission rate enhancement for all the PMMA coated NDs.

### Numerical Analysis

Numerical simulations were performed using COMSOL and RSoft packages for the orthogonal and parallel orientations of the dipole relative to the Si surface. The thickness *t* and refractive index *n*_*PMMA*_ of the PMMA film and the average ND radius *z *= 30 nm were used as the representative parameters for modeling.

The optical emission characteristics of ND encapsulated NV^−^ centers coated with a PMMA matrix were computationally investigated using the finite element method (FEM) implemented in COMSOL MULTIPHYSICS and Finite Difference Time Domain (FDTD) in RSoft. The study was carried out in two steps. In the first step, ND on the Si substrate was considered, as illustrated in Fig. 2(a). In the second step, a layer of PMMA was introduced in the model as illustrated in Fig. 2(b). The geometry consists of a Si substrate coated with 111 nm thick PMMA and air cladding. The refractive indices of the Si and PMMA used in the simulations were *n*_*Si*_ = 3.875 + 0.0111*i* and *n*_*PMMA*_ = 1.48 respectively. A perfectly matched layer was used at the boundaries of the computational domain to absorb outgoing radiation. Two extreme polarizations of the dipole i.e., orthogonal and parallel relative to the Si substrate were considered.

Table 2 shows the results of simulations which confirm the emission rate enhancement in the range of 1.18 to 3.58 using FEM and 1.91 to 2.85 using FDTD, for orthogonal and parallel polarizations respectively. These measurements were performed for the experimental thickness of 111 nm for PMMA coating. The two ranges are in good agreement with the experimental range of enhancement defined by Table 1.

**Figure 11:**
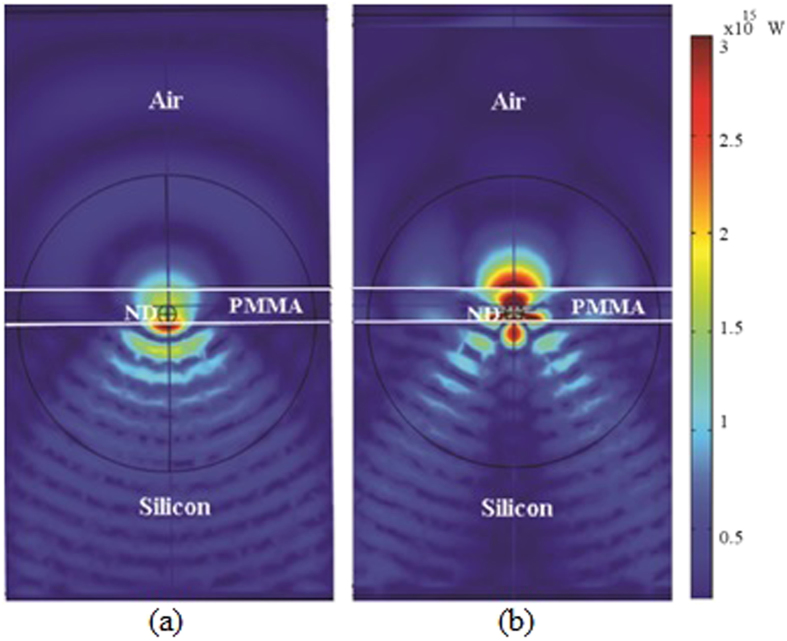
Electric field distribution emitted by dipole in the ND for (**a**) parallel and (**b**) orthogonal dipole polarizations. The ND is placed on a semi-infinite Si substrate and coated with 111 nm thick PMMA film.

The electric field distribution emitted by a ND coated in the PMMA is shown in Fig. 11 (a,b) for parallel and orthogonal components.

It can be seen that the majority of the electric field distribution is present in the Si due to its high refractive index. This is the case before coating the PMMA on the Si substrate. This clearly reveals that the enhancement in the emission after coating the PMMA is due to lifetime reduction of the emitter in ND and is not due to any increased directionality of the emitted radiation pattern in agreement with the experimentally observed data.

**Figure 12:**
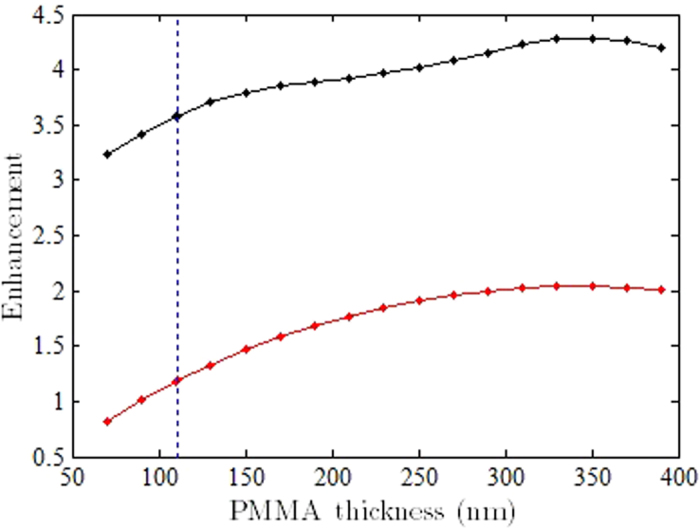
Enhancement of the total mean power radiated for both orthogonal (red) and parallel (black) polarizations when the ND is coated with varying thicknesses of PMMA. The dotted blue line indicates the experimental thickness and the associated orthogonal and parallel polarisation enhancements.

We also numerically evaluated the effect of varying PMMA thickness on the emission enhancement. The graph of Fig. 12 plots the enhancement as a function of PMMA thickness. Figure 12 shows that for the parallel and orthogonal polarizations, the enhancement varies within a range of 0.8 to 4.2 with the variation in PMMA film thickness. This means that experimentally changing the thickness of PMMA for the sample will increase or decrease the enhancement ratio in a range spanned by the two curves. The dotted blue line indicates the experimental value of thickness *t *= 111 nm investigated above.

An important point to note here is that the position *z* of the dipole with respect to the substrate and its orientation inside the ND are not precisely known. From AFM measurements performed for 10 NDs, *z* can lie anywhere between 0 nm and an average maximum of 65 nm with respect to the substrate. In addition the PMMA thickness *t* is also a crucial parameter which governs the emission rate of the dipole. Consequently *z* is changing for all the NDs listed in Table 1, which gives us a range of different enhancement values.

## Conclusions

We report the radiative emission rates and lifetime ratios of the polymer coated NDs with respect to air. Reduced lifetimes and brighter emission rates are observed for all investigated single NV^−^ centers. The increase in emission rates is consistent with numerical simulations performed. Enhancement is interpreted in terms of changing the local electromagnetic environment seen by the emitted photons, i.e. the refractive index of the upper interface from 1.0 (air) to 1.48 (PMMA). The main contribution to the enhancement comes from the presence of thin polymer film in the close proximity between objective and ND.

The marked Si substrate enables identification of the same centers before and after coating. The enhancement comes solely from ND coated with polymer that has a higher refractive index than air. The Si substrate decreases the overall enhancement as the refractive index contrast between the upper and lower regions are decreased with the coating. Consequently, reflectivity from the Si substrate is reduced. If NDs are only coated within a polymer film, according to our FDTD calculations, the enhancement ratio is even larger without the Si substrate. Coating ND with a height of 60 nm within a 162 nm thick polymer layer results in an 18% and 7% enhancement in the orthogonal and parallel dipole polarizations respectively. Applying the same condition to a dipole in free-space, without ND encapsulation results in the reduction of emission. Therefore the enhancement is due to the reduction of the refractive index difference between the diamond nanoparticle and surrounding environment.

Our current study concludes that the radiative enhancement PMMA/air, depends mainly on the refractive index of coating polymer and its thickness. Our previous results on silk coated NDs, with the refractive index of *n *= 1.54, suggest that a shift to higher index polymer coating further increases the emission^6^. The refractive index contrast between PMMA, diamond and Si substrate, the NV^−^ dipole’s orientation and the radius of the encapsulating ND are key parameters in determining the emission properties of NV^−^ in a structured environment. A variation in the emission enhancement for 10 different emitters is reported which is associated to different ND heights and the orientation of the NV^−^ dipole inside the ND. The enhancement in emission observed experimentally is supported by FEM and FDTD models.

## Experimental Section

*Nanodiamonds*: Commercially available non-detonation NDs (NaBond) were used as received with an average diameter of 60 nm. The percentage of NDs containing naturally occurring single NV^−^ centers is approximately 2% of the NDs which fluoresce.

*FIB marking and AFM*: Marker milling was done using Ga^+^ ions accelerated with a 30 keV voltage and a 7 nA current. This produced marker pattern, approximately 3 μm deep and enabled the detection of emitters with the confocal microscope.

*Ellipsometry*: The refractive index of the caoted PMMA thin film, *n*_*PMMA*_ and its thickness, *t* was measured experimentally with J.A.Woollam M-2600DI SE. The SE response curves were fitted with a computer model to find *n*_*PMMA*_ and *t* that best matches the curves.

*Confocal microscopy*: Confocal fluorescence scanning was performed using a customized, built in-house, confocal microscope. The spontaneous emission properties of the fluorescing centers were studied with a continuous wave laser illuminating the sample at a wavelength of *λ *= 532 nm. The fluorescence of the NDs was collected with a 100×, 0.95 NA microscope objective with an in-plane spatial resolution of approximately 400 nm. The fluorescent emitters on the scan were inspected with a Hanbury-Brown and Twiss interferometer for the presence of single photon emission indicating individual NV^−^ centers. An SPS emits one photon at a time with zero probability of multi-photon emission^1^. From each emitter on the confocal scan of Fig. 5, photons were collected and split in a 50:50 multimode fibre splitter and routed to two single photon avalanche diodes. An electronic delay was inserted in one of the detection channels to introduce a time delay *∆t* between the events at the two detectors^31^.

The coincident photon counts at the two detectors were recorded as a function of *∆t* between simultaneous photon detection events. A dip in the coincident counts with the decrease in the delay time characterizes a single photon emitter. The single photon characteristics of the recorded data were studied using the intensity autocorrelation function *g*^*(2)*^ *(∆t)*. For an ideal single photon emitter, the *g*^*(2)*^ *(∆t)* function for the two outputs is *g*^*(2)*^ *(0)* = 0. For all *∆t* > *τ*, *g*^*(2)*^ *(∆t)* > *g*^*(2)*^ *(0)*, because after emission of a single photon, the emitter needs to be excited again before a second photon is emitted. Here *∆t *= *τ* denotes the lifetime of the emitter^32^.

## Additional Information

**How to cite this article**: Khalid, A. *et al.* Lifetime Reduction and Enhanced Emission of Single Photon Color Centers in Nanodiamond via Surrounding Refractive Index Modification. *Sci. Rep.* **5**, 11179; doi: 10.1038/srep11179 (2015).

## Figures and Tables

**Figure 1:**
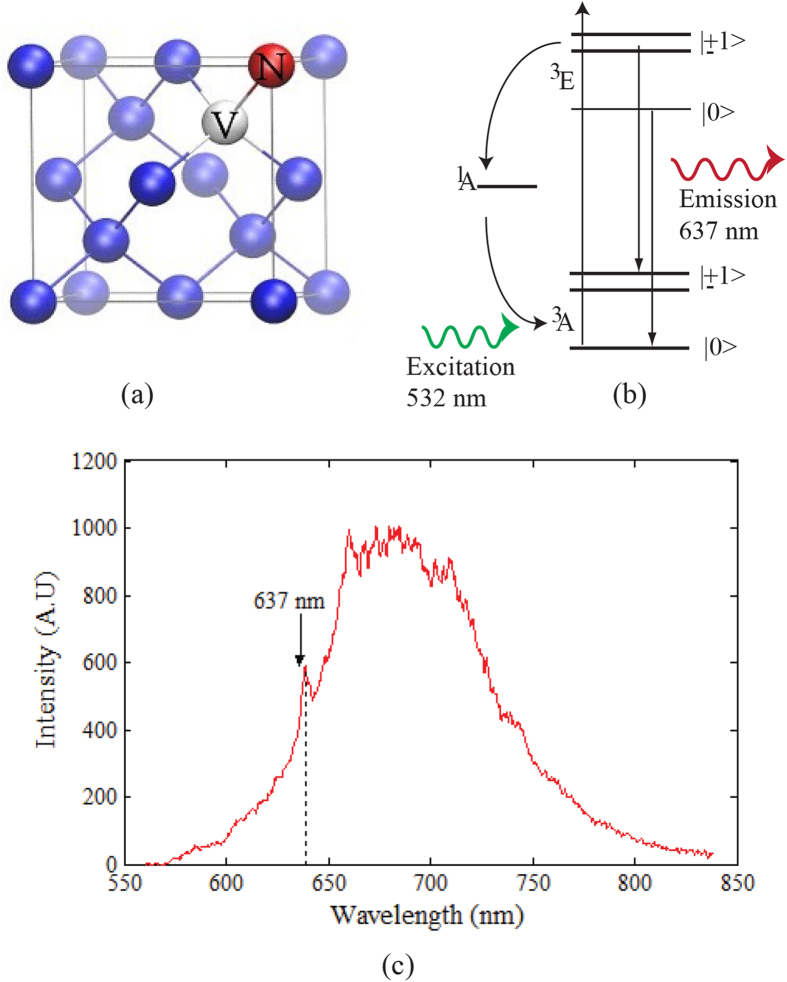
(**a**) Schematic of the NV^−^ center in a diamond lattice showing the vacancy (V) and the substitutional nitrogen atom (red). (**b**) Energy level diagram of NV^−^ center^8^. (**c**) Photoluminescence (PL) spectrum of NV^−^ center at room temperature with a zero phonon line at 637 nm and phonon side band from 650–750 nm is shown. Arrows indicate the peaks positions of the NV^−^ zero phonon line and phonon side band.

**Table 1:** Experimental data for ten NV^−^ centers with single photon emission including lifetime before and after polymer coating, lifetime reduction and emission rate enhancement.

**No**	**Lifetime - no coating τ_b_ [ns]**	**Lifetime - with coating τ_a_ [ns]**	**Lifetime reduction τ_b_/τ_a_**	**Emission enhancement**
1	15.98 ± 3.30	9.66 ± 3.95	0.60 ± 0.27	1.50 ± 0.10
2	14.02 ± 4.61	8.32 ± 1.41	0.59 ± 0.21	2.10 ± 0.30
3	22.11 ± 7.74	11.03 ± 2.72	0.50 ± 0.21	1.30 ± 0.10
4	19.17 ± 2.63	11.10 ± 1.50	0.57 ± 0.11	1.90 ± 0.20
5	23.74 ± 2.95	16.39 ± 2.04	0.69 ± 0.12	1.50 ± 0.10
6	13.40 ± 1.50	7.70 ± 0.50	0.57 ± 0.07	1.50 ± 0.40
7	10.57 ± 0.76	9.47 ± 0.45	0.89 ± 0.07	1.10 ± 0.10
8	12.13 ± 1.23	9.63 ± 1.11	0.79 ± 0.12	1.40 ± 0.10
9	29.43 ± 4.06	14.04 ± 0.69	0.48 ± 0.07	2.10 ± 0.20
10	28.82 ± 3.62	18.80 ± 1.35	0.65 ± 0.09	1.90 ± 0.20

**Table 2:** Numerical results for emission enhancement calculated using two numerical methods, finite element method (FEM) and finite-difference time domain (FDTD).

**Method**	**Emission enhancement** ***E=P***_***P***_***/P***_***A***_	
	**Polarization**	
	orthogonal	parallel
FEM	1.18	3.58
FDTD	1.91	2.85

The diameter of the ND and the thickness of the PMMA film was taken as 60 nm and 111 nm respectively. Polarization is defined relative to the Si surface.
